# Supplementary material for: Development of a denoising convolutional neural network-based algorithm for metal artifact reduction in digital tomosynthesis for arthroplasty: A phantom study
Source: PLoS One. 2019 Sep 13;14(9):e0222406. doi: 10.1371/journal.pone.0222406 (PMC6743787; doi:10.1371/journal.pone.0222406)
Supplement: Attachment — Submitted filename: rebuttal_letter.pdf [file pone.0222406.s001.pdf]

MS Number: PONE-D-19-15441

MS Title: Development of a denoising convolutional neural network-based algorithm for metal artifact reduction in digital tomosynthesis for arthroplasty: A phantom study

PLOS ONE Academic Editor

Dear Dr. Li Zeng

Thank you in advance for your kind consideration of this paper. I attach here our revised manuscript, as well as a point-by-point response to the reviewer's comments.

We feel that the revised manuscript is a suitable response to the comments, and is significantly improved over the initial submission.

Sincerely yours.

Journal requirements:

When submitting your revision, we need you to address these additional requirements.

1. Please ensure that your manuscript meets PLOS ONE's style requirements, including those for file naming.

Response: We reviewed the submission rules again before submitting the revised manuscript. Then, we made a revised manuscript according to the submission rules. We are confident that the revised manuscript is in accordance with the submission rules.

2. Please ensure that you have fully discussed how the present study advances on your previous work in this area. Please ensure that you discuss how your work relates to, and advances upon, the following publication:

"Development of a novel algorithm for metal artifact reduction in digital tomosynthesis using projection-based dual-energy material decomposition for arthroplasty: A phantom study"

Response: We examined the image quality improvement method aiming at metal artifact reduction (MAR) in tomosynthesis imaging and reported its usefulness. Using dual-energy (DE) material decomposition published in 2018, DEMDRA (Gomi et.al. Development of a novel algorithm for metal artifact reduction in digital tomosynthesis

using projection-based dual-energy material decomposition for arthroplasty: A phantom study. Phys.Med. 2018) achieves an effective MAR compared to single energy imaging. DEMDRA is the most useful MAR method at present, and based on this method, in order to pursue further improvement of image quality and universality, this research has developed a method that combines DE and deep learning. In this research, virtual monochromatic (VM) image by DE is used as data for learning. The difference between this study and DEMDRA is that it does not use deep learning and material decomposition. DE cannot be acquired by all tomosynthesis devices, but there is a peculiarity. For this reason, we have started developing methods using deep learning to realize the universality of MARs in other devices. As a result of comparing this research with the conventional method, it has been clarified that it is effective in improving the longitudinal MAR and the reproducibility in the slice plane. This research is not the same as the conventional method, and it is novelty that the universal system was constructed by combining the methods useful for MAR. We are convinced that we have not violated any of the ethical guidelines in relation to the content of this study.

<u>Response to the comments of Editor</u>:

Comment: Please use the space provided to explain your answers to the questions above. You may also include additional comments for the author, including concerns about dual publication, research ethics, or publication ethics. (Please upload your review as an attachment if it exceeds 20,000 characters)

Response: Please refer to the author's response to the request from journal.

<u>Response to the comments of Reviewer #1</u>:

We wish to express our appreciation to the reviewer for offering insightful comments, which have helped us improve the paper significantly.

Comment: The authors are developing a novel denoising convolutional neural network metal artifact reduction hybrid reconstruction (DnCNN-MARHR) algorithm for decreasing metal objects in digital tomosynthesis (DT) for arthroplasty employing projection data. The DnCNN-MARHR algorithm based on a training network (mini-batch stochastic gradient descent algorithm with momentum) to estimate residual reference and object images using projection data and subtract the estimated residual images from the object images, involving hybrid and subjectively reconstructed image usage (back

projection and maximum likelihood expectation maximization.
However, the proposed methodology is not new. The paper simply combines several preexisting and widely available techniques, such as, convolutional neural network, residual learning, mini-batch stochastic gradient descent algorithm, back projection maximum likelihood expectation maximization. In this context, what are the methodological and algorithmic contributions of the work to the research community?
Response: We appreciate the reviewer's comment on this point. The following is a description of our approach to the method proposed in this study. In this research, virtual monochromatic (VM) image by DE is used as data for learning. The difference between this study and DEMDRA is that it does not use deep learning and material decomposition. DE cannot be acquired by all tomosynthesis devices, but there is a peculiarity. For this reason, we have started developing methods using deep learning to realize the universality of MARs in other devices. As a result of comparing this research with the conventional method, it has been clarified that it is effective in improving the longitudinal MAR and the reproducibility in the slice plane. This research is not the same as the conventional method, and it is novelty that the universal system was constructed by combining the methods useful for MAR.

Comment: Over all the paper, I'm not sure the purpose of this paper is denoising or metal artifacts reduction.
Response: We agree that this point requires clarification, and have added the following text to the Introduction section.
To support this basis, DnCNN is designed primarily to remove noise from the image. It comprises of a built-in deep feedforward CNN. However, as the DnCNN uses a residual learning method, it is also possible to train the DnCNN architecture to reduce artifacts. In the residual learning method, effects as the MAR method can be expected because the residual image is estimated by learning in the DnCNN network.
(Remark_1)

Comment: The structure of the article is very confusing. For instance, the equation of the evaluation criteria (artifact index) is in the Optimization parameters section and the other two evaluation criterias are in the Evaluation section.
Response: We agree that this point requires clarification, and have revised the text to the Evaluation and Results section. Descriptions of optimization parameters were integrated into the Evaluation section, and parameter evaluation results were integrated into the Results section.

(Remark_2)

Comment: The methods section is badly written. Many important aspects in the methodology are left unexplained in the article. The authors state to use low-energy projection image (PL) as input image and VM X-ray image as reference image for training workflow (Page 8, Line 159-160). There is no information on how many total data sets and how may data sets were allocated each to training, validation and testing.
Response: We agree that this point requires clarification, and have added the following text to the Generation of reference projection images and Evaluation section:
[Generation of reference projection images section]
Thirty-seven reference images (VM X-ray projection image) corresponding to the corrected input image pairs were randomly selected as the training set from the original projection data set (total original projection data set: 74).
(Remark_3)

[Evaluation section]
We set the patch size as 32 × 32, and crop [mini-batch size] × [maximum number of iterations: epoch × number of projection (37)] patches to train the model.
(Remark_3)

Comment: It is very confusing in how to generate the reference projection images VM X-ray image? What is the meaning of the Ft, Fw and Ff in equation (5), how to determine the value of Ft, Fw and Ff? And why VM X-ray image can be the reference projection images for traning the DnCNN, there's still artifacts in this image in my opinion.
Response: We agree that this point requires clarification, and have added the following text to the Generation of reference projection images section:
In the MAR, it has been reported that DEMDRA applying material decomposition is the most effective for MAR [1]. However, DEMDRA has to apply DnCNN to projection data separated into a plurality of material decompositions. Then, it may be difficult to maintain the residual accuracy with the polychromatic projection data to be compared. Conversely, VM X-ray imaging can learn residuals with high accuracy by using a single projection datum; thus an effective MAR can be expected. Therefore, the VM X-ray image was used as a reference image in this study.

1. Gomi et.al. Development of a novel algorithm for metal artifact reduction in digital tomosynthesis using projection-based dual-energy material decomposition for arthroplasty: A phantom study. Phys.Med. 2018;53:4-16.

(Remark_4)

The theoretical mass attenuation coefficient and linear attenuation coefficient curve shown in Fig 4 were calculated using the local density and area density of each material. These are generated by inputting the chemical compositions of the titanium alloy, foam cortical shell, and water shown in Table 1 into the XCOM program developed by Berger and Hubbell [2]. Finally, for the projection space decomposition approach, the following process was used to generate material decomposition images for titanium alloy, foam cortical shell, and water.

Equations (2) and (3) were used to calculate values for P_{L_t}, P_{L_w}, P_{L_f}, P_{H_t}, P_{H_w}, and P_{H_f} as simulated attenuation intensities of these materials at the two energy levels. These values were then used to construct a sensitivity matrix, and the material fractions were obtained from the inverse of this matrix, as shown in equation (5):

$$\begin{bmatrix} F_t \\ F_w \\ F_f \end{bmatrix} = \begin{bmatrix} P_{L\_t} & P_{L\_w} & P_{L\_f} \\ P_{H\_t} & P_{H\_w} & P_{H\_f} \\ 1.0 & 1.0 & 1.0 \end{bmatrix}^{-1} \begin{bmatrix} XDTS_{EL} \\ XDTS_{EH} \\ 1.0 \end{bmatrix} \quad (5)$$

$$F_t P_{L\_t} + F_w P_{L\_w} + F_f P_{L\_f} = XDTS_{EL}$$

$$F_t P_{H\_t} + F_w P_{H\_w} + F_f P_{H\_f} = XDTS_{EH}$$

$$F_t + F_w + F_f = 1.0$$

2. Berger M, Hubbell J. Photon cross sections on a personal computer. Gent Radiat Res. 1987:1-28. doi: 10.2172/6016002.

(Remark_4)

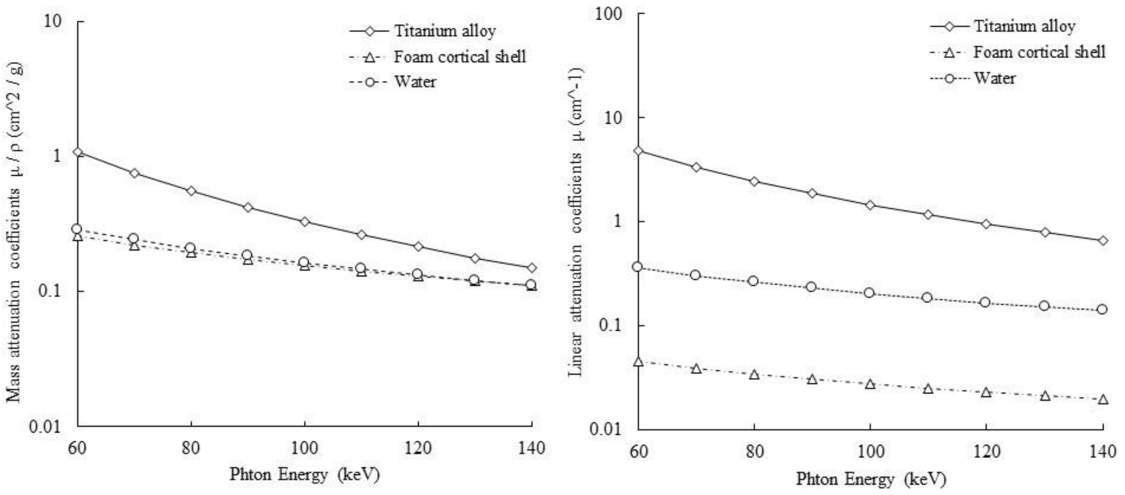

Fig 4. The linear attenuation and mass attenuation coefficients of a foam cortical shell, titanium alloy, and water with respect to the photons. Based on the linear attenuation coefficient map, each energy image of virtual monochromatic X-ray processing was created.

(Remark_4)

Comment: In fig 2, I cannot find the difference between the original image (PL) and artifact reduced projection image (Cor_img) from the DnCNN step. I suggest that the authors can directly use the original image (PL) and the equation (11) to obtain the final MAR image and make a comparison between this MAR image and DnCNN-MARHR image, I think the difference in the image quality between this MAR image and DnCNN-MARHR image is small.

Response: We agree that this point requires clarification, and have added the following text to the Abstract, Evaluation and Results section:

[Abstract]

In addition, comparison of the difference (mean square error) between DnCNN-MARHR and the conventional algorithm resulted in the smallest VM.

(Remark_5)

[Evaluation section]

To compare the difference between DnCNN-MARHR and the conventional algorithm, the difference was evaluated using differential images and mean square error (MSE) in the in-focus plane.

(Remark_5)

[Evaluation section]

Mean square error (MSE)

The MSE of identified in-focus plane image is given as below:

$$MSE = \frac{1}{mn}\sum_{i=0}^{m-1}\sum_{j=0}^{n-1}\left[K_{ref}(i,j) - V_{obj}(i,j)\right]^2$$

where, $K_{ref}(i,j)$ is the (i,j) th entry of a DnCNN-MARHR image and $V_{ref}(i,j)$ is the (i,j) th entry of an each conventional algorithm image.

(Remark_5)

[Results section]

Comparison of the difference between DnCNN-MARHR and the conventional algorithm resulted in the smallest VM (Table 2).

(Remark_5)

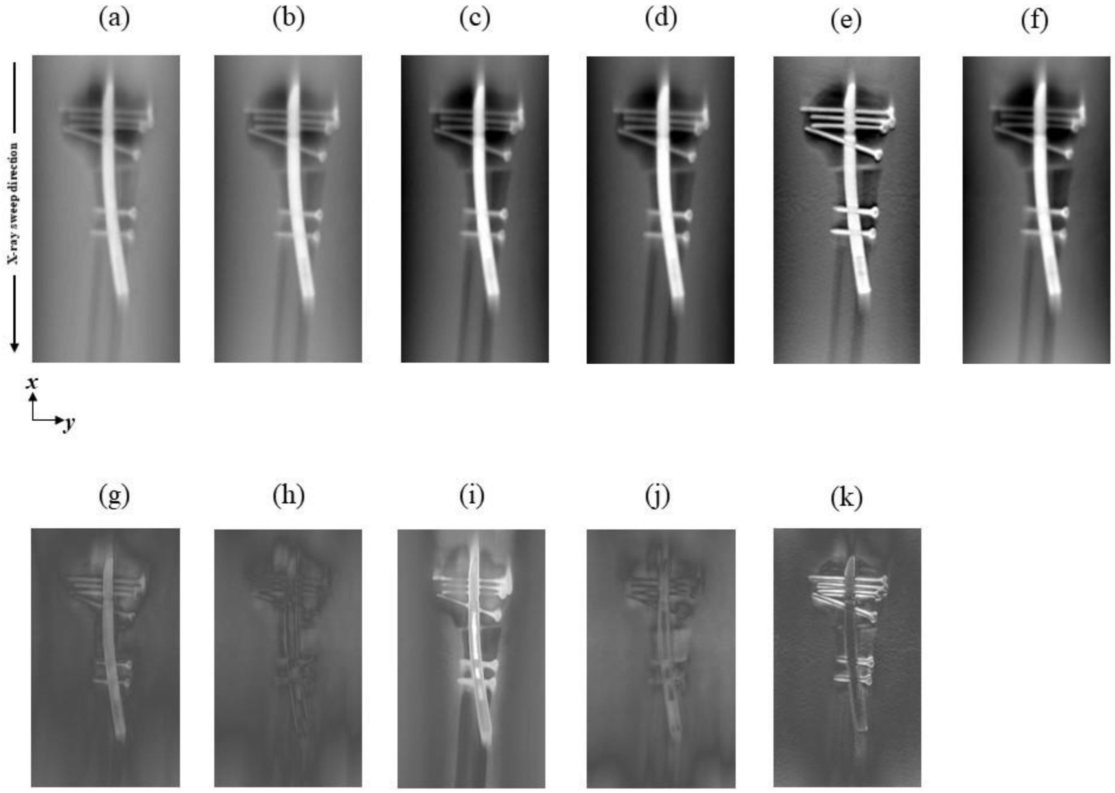

Fig 8. Comparisons among the denoising convolutional neural network metal artifact reduction hybrid reconstruction (DnCNN-MARHR) algorithm and the traditional reconstruction algorithms with metal artifact reduction (MAR) processing in the in-focus plane. (DnCNN-MARHR (a), 0.8029–0.9119; DEMDRA (b), 0.9597–0.9914; dual-energy virtual monochromatic [VM]-MAR [140 keV] (c), 0.9487–0.9934; maximum likelihood expectation maximization [MLEM]-MAR [70 kV] (d), 0.9411–0.9983; filtered back projection [FBP, kernel: Shepp & Logan]-MAR [70 kV] (e), 0.5676–0.6117; simultaneous algebraic reconstruction technique-total variation [SART-TV]-MAR [140 kV] (f), 0.7577–0.8184, difference between DnCNN-MARHR and DEMDRA (g), 0–0.1047; difference between DnCNN-MARHR and [VM]-MAR [140 keV] (h), 0–0.1047; difference between DnCNN-MARHR and [MLEM]-MAR [70 kV] (i), 0–0.1047; difference between DnCNN-MARHR and [SART-TV]-MAR [140 kV] (j), 0–0.1047; difference between DnCNN-MARHR and [FBP, kernel: Shepp & Logan]-MAR [70 kV] (k), 0–0.1047) The display variety of the prosthetic phantom is changed to make visual comparison of the contrast and background gray levels. The X-ray source is moved along the image vertically. In the displayed areas, the artifact indices are determined.

(Remark_5)

Table 2 Mean square error (MSE) between each reconstruction algorithm.

	**MSE**
**DnCNN-MARHR between DEMDRA**	3.8048e-05
**DnCNN-MARHR between VM with MAR(140keV)**	3.5683e-05
**DnCNN-MARHR between MLEM with MAR(70kV)**	3.7185e-04
**DnCNN-MARHR between SART-TV with MAR(140Kv)**	9.6985e-05
**DnCNN-MARHR between FBP with MAR(70Kv)**	1.0318e-04

Results of comparison of the mean square error (MSE) between the DnCNN-MARHR image and each MAR image. (Comparison image is in-focus plane).

(Remark_5)

Thank you again for your comments on our paper. I trust that the revised manuscript is now suitable for publication.

Response to the comments of Reviewer #2:

We wish to express our appreciation to the reviewer for offering insightful comments, which have helped us improve the paper significantly.

Comment: As far as I know, the material decomposition algorithm using dual-energy imaging technique needs a known material phantom to build the relationship between the projection (attenuation) and the material property in the calibration process. For the testing process, the projection of the unknown object with low and high energy was converted to the basis images with known materials in the projection domain. The virtual

monochromatic images (VMIs) are generated by using the basis image and the attenuation coefficient according to the linear relation of the attenuation coefficient combination. Do you use the calibration phantom? Please provide more information on the content.

Response: We agree that this point requires clarification, and have added the following text to the Generation of reference projection images section:

In the MAR, it has been reported that DEMDARA applying material decomposition is most effective for MAR [1]. However, DEMDRA has to apply DnCNN to projection data separated into a plurality of material decompositions. Then, it is possible that it may be difficult to maintain the residual accuracy with the polychromatic projection data to be compared. On the other hand, VM X-ray image can learn residuals with high accuracy by using a single projection data, and an effective MAR can be expected. Therefore, VM X-ray image was used as a reference image in this study.

1. Gomi et.al. Development of a novel algorithm for metal artifact reduction in digital tomosynthesis using projection-based dual-energy material decomposition for arthroplasty: A phantom study. Phys.Med. 2018;53:4-16.

(Remark_4)

The theoretical mass attenuation coefficient and linear attenuation coefficient curve shown in Fig 4 were calculated using the local density and area density of each material. These are generated by inputting the chemical compositions of the titanium alloy, foam cortical shell, and water shown in Table 1 into the XCOM program developed by Berger and Hubbell [2]. Finally, for the projection space decomposition approach, the following process was used to generate material decomposition images for titanium alloy, foam cortical shell, and water.

Equations (2) and (3) were used to calculate values for P_{L_t}, P_{L_w}, P_{L_f}, P_{H_t}, P_{H_w}, and P_{H_f} as simulated attenuation intensities of these materials at the two energy levels. These values were then used to construct a sensitivity matrix, and the material fractions were obtained from the inverse of this matrix, as shown in equation (5):

$$\begin{bmatrix} F_t \\ F_w \\ F_f \end{bmatrix} = \begin{bmatrix} P_{L\_t} & P_{L\_w} & P_{L\_f} \\ P_{H\_t} & P_{H\_w} & P_{H\_f} \\ 1.0 & 1.0 & 1.0 \end{bmatrix}^{-1} \begin{bmatrix} XDTS_{EL} \\ XDTS_{EH} \\ 1.0 \end{bmatrix} \quad (5)$$

$$F_t P_{L\_t} + F_w P_{L\_w} + F_f P_{L\_f} = XDTS_{EL}$$

$$F_t P_{H\_t} + F_w P_{H\_w} + F_f P_{H\_f} = XDTS_{EH}$$

$$F_t + F_w + F_f = 1.0$$

2. Berger M, Hubbell J. Photon cross sections on a personal computer. Gent Radiat Res. 1987:1-28. doi: 10.2172/6016002.

(Remark_4)

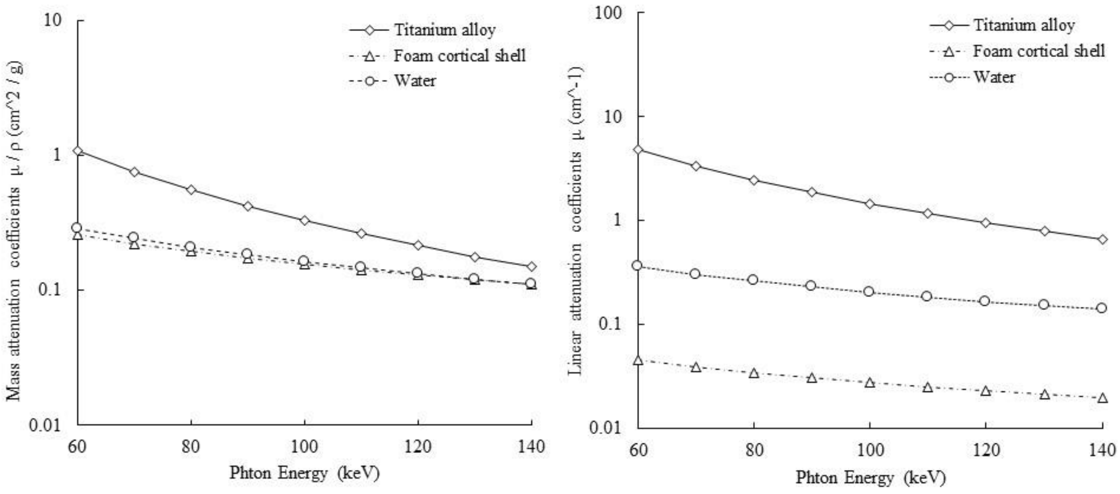

Fig 4. The linear attenuation and mass attenuation coefficients of a foam cortical shell, titanium alloy, and water with respect to the photons. Based on the linear attenuation coefficient map, each energy image of virtual monochromatic X-ray processing was created.

(Remark_4)

Comment: The DnCNN-MARHR algorithm is the major development of the study. And the Eq.(7) is the core to calculate the residual projection image. Please add more explanations or references.

Response: We agree that this point requires clarification, and have added the following text to the DnCNN-MARHR section:

Equation is a two-layer feed-forward CNN. The CNN architecture further generalizes one-stage trainable nonlinear reaction diffusion (TNRD) [1, 2] from three aspects:

(1) Replacing the influence function with ReLU to ease CNN training;

(2) Increasing the CNN depth to improve the capacity in modeling image characteristics;
(3) incorporating batch normalization to boost the performance.

The connection with one-stage TNRD provides insights into the use of residual learning for CNN-based image restoration [3].

1. Chen Y, Yu W, Pock T. On learning optimized reaction diffusion processes for effective image restoration. IEEE Conference on Computer Vision and Pattern Recognition. 2015:5261–9.
2. Chen Y, Pock T. Trainable nonlinear reaction diffusion: A flexible framework for fast and effective image restoration. IEEE transactions on Pattern Analysis and Machine Intelligence. 2016;39(6):1256-72.
3. Zhang K, Zuo W, Chen Y, Meng D, Zhang L. Beyond a gauaaian denoiser: residual learning of deep CNN for image denoising. IEEE Trans Image Process. 2017;26(7):3142 – 55.

(Remark_6)

Comment: The specification of the computing environment? How long does it take for training and testing process? Iteration number of different image reconstruction? The differences in the calculation time cost for DnCNN-MARHR algorithm and other methods.

Response: We agree that this point requires clarification, and have added the following text to the Results, Evaluation, and DnCNN-MARHR section:

[Results section]

Training was implemented by MATLAB on a CPU (Intel(R) Xeon(R) E5-2620 v4, 2.10GHz, 2 processor) and four GPU (NVIDIA Tesla K40c, 12GB) systems. The network required approximately 15 h for training. The reconstruction time was approximately 30 minutes for DnCNN-MARHR (not included in the training network) and DEMDRA approximately 20 minutes for MLEM and SART-TV, and approximately 2 minutes for FBP.

(Remark_7)

[Evaluation section]

The previously reported iteration numbers (DEMDRA, VM, MLEM; 30, SART-TV; 10) were applied to these conventional algorithms for MAR [1]. An iteration number for TV minimization of 100 and length per gradient-descent step of 50 were considered optimal parameters for SART-TV [1]. DnCNN was evaluated using the application image of optimization parameters.

1. Gomi T, Sakai R, Goto M, Hara H, Watanabe Y, Umeda T. Evaluation of digital tomosynthesis reconstruction algorithms used to reduce metal artifacts for arthroplasty: A phantom study. Phys Med. 2017;42:28-38.

(Remark_7)

[DnCNN-MARHR section]

X^T is back projection, X is multiplication by the system matrix, T (superscript) is the matrix response, u is the number of iterations, $MLEM_{-DnCNN}$ is the MLEM [iteration (convergence): 30] image from artifact-reduced projection, BP_{-DnCNN} is the back-projection image from artifact-reduced projection, $MLEM_{-DnCNN}$ is the DnCNN-MARHR image, and w is the weighting coefficient.

(Remark_7)

Comment: What is the image size of the projection? Does it have any image pre-processing on the projections?

Response: We agree that this point requires clarification, and have added the following text to the DE-DT system and Generation of reference projection images section:

[DE-DT system section]

37 projection images (1024 × 1024 matrix)

(Remark_8)

[Generation of reference projection images section]

Thirty-seven reference images (VM X-ray projection image) corresponding to the corrected input image pairs were randomly selected as the training set from the original projection data set (total original projection data set: 74).

(Remark_3)

Comment: DEMDRA has the lowest AI value. Why not use DEMDRA image to be the reference image of the training network?

Response: We agree that this point requires clarification, and have added the following text to the Generation of reference projection images section:

In the MAR, it has been reported that DEMDARA applying material decomposition is most effective for MAR [1]. However, DEMDRA has to apply DnCNN to projection data separated into a plurality of material decompositions. Then, it is possible that it may be difficult to maintain the residual accuracy with the polychromatic projection data to be compared. On the other hand, VM X-ray image can learn residuals with high accuracy by

using a single projection data, and an effective MAR can be expected. Therefore, VM X-ray image was used as a reference image in this study.

1. Gomi et.al. Development of a novel algorithm for metal artifact reduction in digital tomosynthesis using projection-based dual-energy material decomposition for arthroplasty: A phantom study. Phys.Med. 2018;53:4-16.

(Remark_4)

Comment: In generally, the metal artifacts could be reduced by increasing tube voltage for 3D X-ray imaging. (M.-J.Lee et al., “Overcoming artifacts from metallic orthopedic implants at high-field-strength MR imaging and multi-detector CT,” Radiographics, vol. 27, no. 3, pp. 791–803, 2007.) Why do you choose the projection with lower tube voltage to be the input image for DnCNN-MARHR algorithm?

Response: We agree that this point requires clarification, and have added the following text to the DnCNN-MARHR section:

In the presence of high-energy, the difference in the linear attenuation coefficient between the normal tissues becomes narrow, and the contrast tends to decrease. Accordingly, low energy was selected in terms of retention of contrast in normal tissue as well as MAR.

(Remark_9)

Comment: Do you use any additional filters in the low and high tube voltage? Could you provide the energy spectrum of the low and high tube voltage setting?

Response: We used the following inherent and additional filters (fixed to the system) to acquire 70kV and 140kV. No additional filters other than the following are used in this study.

inherent; aluminum [1.1 mm], additional; aluminum [0.9 mm] and copper [0.1 mm]

We agree that this point requires clarification, and have added the following figure (the energy spectrum of the low and high tube voltage) to the Generation of reference projection images section:

Each X-ray spectrum is shown in Fig 3. (Measurement tool: RAMTEC413 Toyo Medic Co., Tokyo, Japan; Detector: CdTe; Channel: 1024 (0.2keV/channel); Measuring method: Compton-scattering measurement [1])

1. Maeda K, Matsumoto M, Taniguchi A. Compton-scattering measurement of diagnostic x-ray spectrum using high-resolution Schottky CdTe detector. Med Phys. 2005;32(6):1542-7. Epub 2005/07/15. doi: 10.1118/1.1921647. PubMed PMID: 16013712.

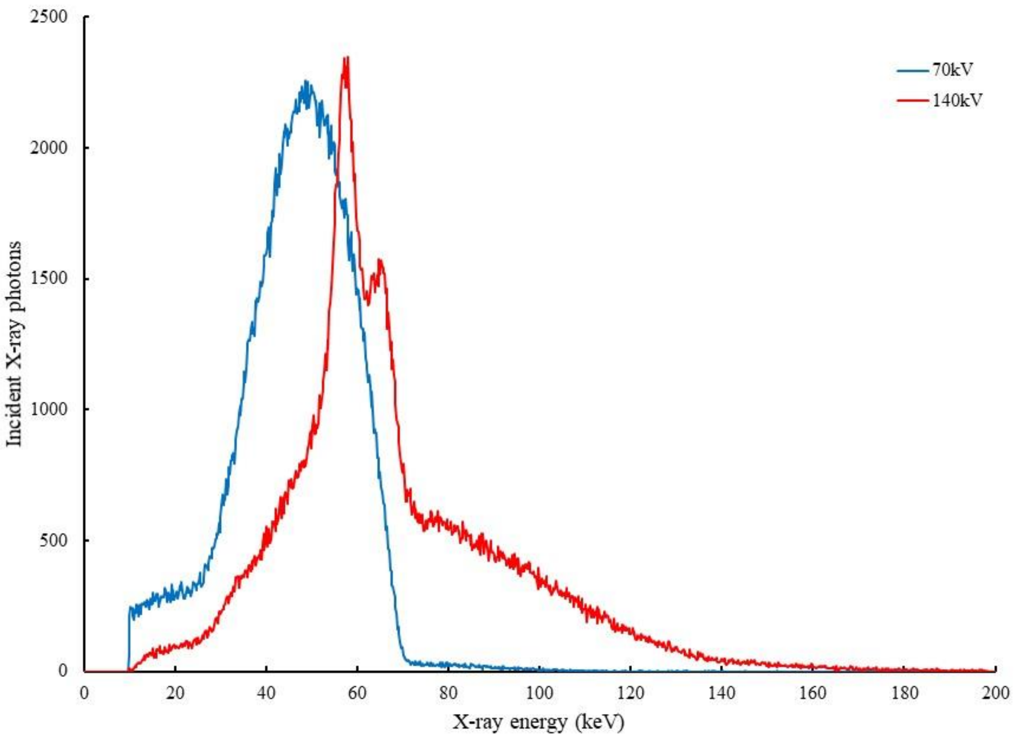

Fig 3. Spectra of the Sonial Vision Safire II tube at 70 and 140 kV potentials. The peaks represent the characteristic lines of the tungsten with rhenium and molybdenum anode and the continuous spectrum is a result of Bremsstrahlung. The mean photon energies are 49 and 80 keV, respectively. (Real filter: inherent; aluminum [1.1 mm], additional; aluminum [0.9 mm] and copper [0.1 mm])

(Remark_10)

Comment: How do you perform the data acquisition of the dual-energy tomosynthesis imaging? Is the imaging with different image parameters in the sequential process?

Response: We agree that this point requires clarification, and have added the following text to the DE-DT system section:

In DE-DT imaging, pulsed X-ray exposures were used with rapid switching between low and high energies.

(Remark_11)

Comment: What is the total slice number of the reconstructed tomosynthesis image? What is the start height of the reconstruction from the detector surface? Where is the focal point during tomosynthesis imaging?

Response: We agree that this point requires clarification, and have added the following text to the DE-DT system section:

total slice number: 50

starting height of the reconstruction from the detector surface: 150mm

The distances between source (focal point)-to-isocenter and isocenter-to-detector were 924 and 1100 mm, respectively.

Figure 1 also added the focal point.

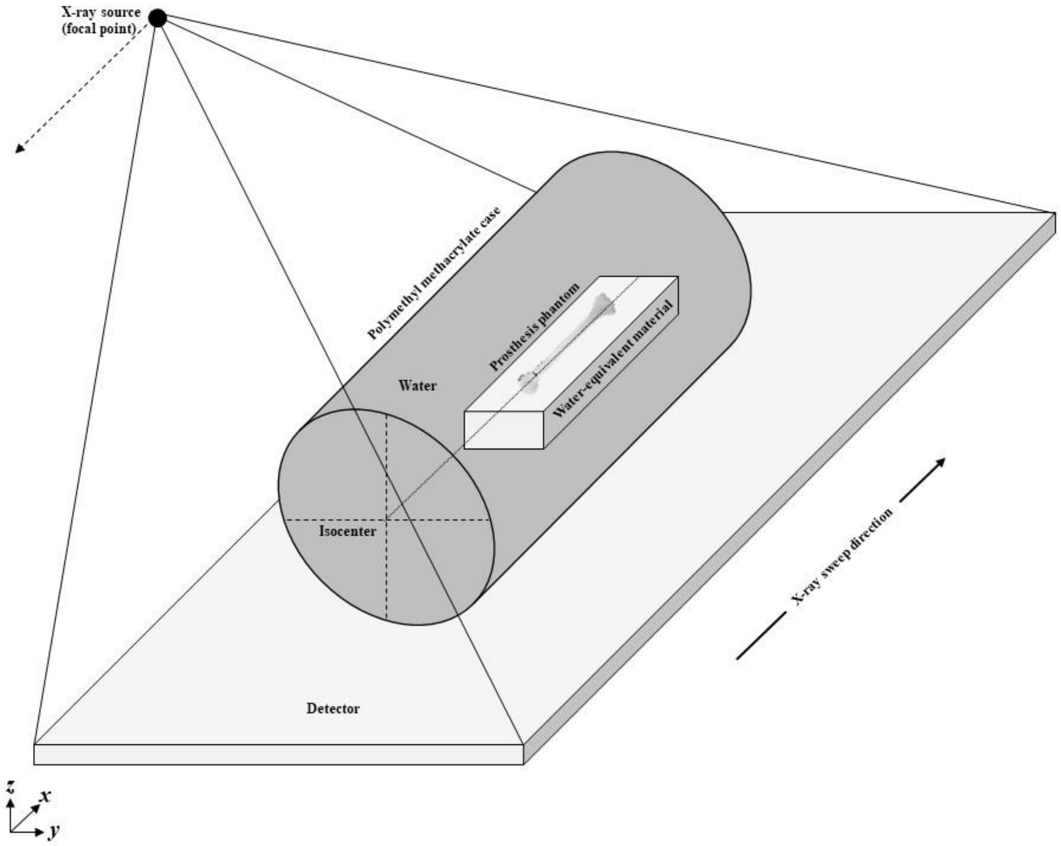

(Remark_12)

Comment: Line 313-315 Could you provide 2D AI surface plot with epochs and weighting coefficients when mini-batch size is 544?

Response: We agree that this point requires clarification, and have added the following figure to the Results section:

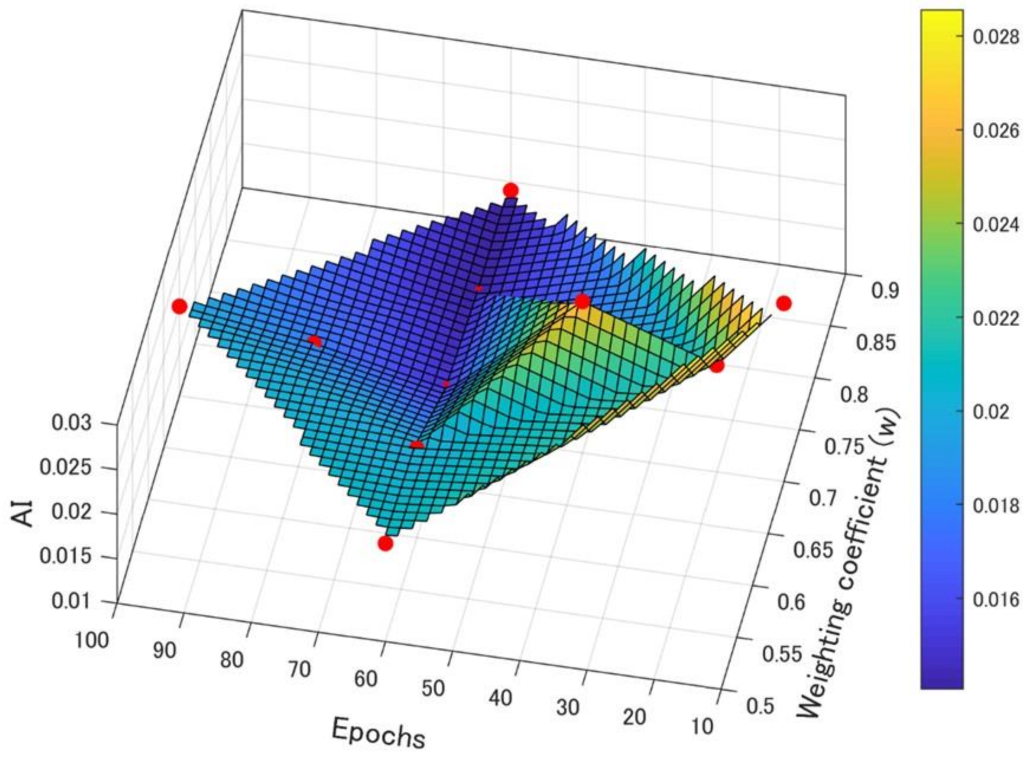

Fig 7. AI surface plot with epochs and weighting coefficients when mini-batch size at 544. The surface plot was processed by cubic linear interpolation.

(Remark_13)

Comment: Line 334-335 In Fig.2, the image of MLEM_DnCNN is the same as the polychromatic MLEM-MAR [70 kV]? And the image of BP_DnCNN is also the same as the polychromatic filtered back projection [FBP] MAR [70 kV]? Why do you combine the image of MLEM_DnCNN and BP_DnCNN? Is it empirical knowledge?

Response: We believe the reviewer is mistaken on this point. The “MLEM_DnCNN” image is a MAR image that is processed by activating learning data to approximate a polychromatic X-ray image to a monochromatic X-ray image, and it is not the same as the “MLEM-MAR” image that is obtained by processing the MAR on a polychromatic X-ray image. Adaptive filtering can be considered as one of the methods for effectively performing MAR by combining both images. For MAR in DT, we report that this adaptive filtering works effectively [1]. Therefore, this adaptive filtering was applied to this study for MAR.

1. Gomi T, Hirano H, Umeda T. Evaluation of the X-ray digital linear tomosynthesis reconstruction processing method for metal artifact reduction. Comput Med Imaging Graph. 2009;33(4):267-74.

Comment: Could you provide the schematic diagram to illustrate the relationship of the in-focus plane and the out-of-plane in the Z-axis direction?

Response: We agree that this point requires clarification, and have added the following text and figure to the DE-DT system section:

The plane locations were the in-focus plane and out-of-plane from the object location. The image reconstructed at the in-focus plane shows that the object is faithfully reconstructed on the focal plane.

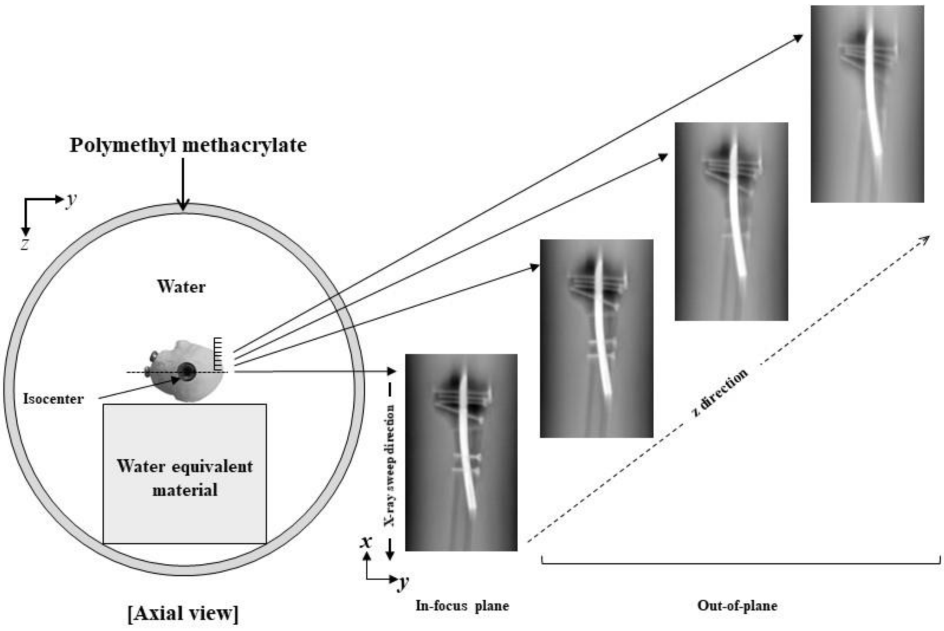

Fig 2. The schematic diagram to illustrate the relationship of the in-focus plane and the out-of-plane in the Z-axis direction. The in-focus plane is not affected by the blur, but the out-of-plane is contains blur. (Remark_14)

Comment: The execute time of DnCNN-MARHR algorithm (not include the training network)?

Response: We agree that this point requires clarification, and have added the following text to the Results section:

Training was implemented by MATLAB on two CPU (Intel(R) Xeon(R) E5-2620 v4, 2.10GHz) and one GPU (NVIDIA Tesla K40c, 12GB) system. The network required approximately 15 h for training. The reconstruction time was 30 minutes for DnCNN-MARHR (not include the training network), DEMDRA and 20 minutes for MLEM, SART-TV, approximately 2 minutes for FBP.

(Remark_7)

Comment: Line 447-448 the imaging parameters case by case? For the object in the different region or the various kind of object (e.g.: materials,…), the proposed algorithm could deal with it?

Response: We agree that information on "applicable in other cases" as the reviewer suggested would be valuable. To make this point clearer, we have added the following to the dissuasion section:

Regarding the success or failure of our proposed method, when it is applied in other cases, the key points are the accuracy involved in the VM X-ray process and the method of extraction of the residual image during learning with high accuracy. For example, an effective MAR may not be expected in cases where normal tissues and artificial bones have a complicated arrangement relation.

(Remark_15)

Comment: Line 137-138 Please normalize the expression of units. –mm µm

Response: In accordance with the reviewer's comment, we have changed "150 × 150 μm^2" to "0.15 × 0.15-mm".

(Remark_16)

Comment: Line 175-176 Please double check the formula correctness of Eq.(2) and Eq.(3). Does it miss the exponential mark?

Response: This error has been corrected in accordance with the reviewer's comment.

$$P_L = \int I_L(E)\exp\left\{-\left(\frac{\mu}{\rho}\right)_t(E)\cdot K_t - \left(\frac{\mu}{\rho}\right)_w(E)\cdot K_w - \left(\frac{\mu}{\rho}\right)_f(E)\cdot K_f\right\}dE \quad (2)$$

$$P_H = \int I_H(E)\exp\left\{-\left(\frac{\mu}{\rho}\right)_t(E)\cdot K_t - \left(\frac{\mu}{\rho}\right)_w(E)\cdot K_w - \left(\frac{\mu}{\rho}\right)_f(E)\cdot K_f\right\}dE \quad (3)$$

(Remark_17)

Comment: Line 238 “Equation (6)”
Response: This error has been corrected in accordance with the reviewer’s comment. (Remark_18)

Comment: Line 245 “Equation (7)”
Response: This error has been corrected in accordance with the reviewer’s comment. (Remark_18)

Thank you again for your comments on our paper. I trust that the revised manuscript is now suitable for publication.
